# No mesh, no problem: an innovative surgical approach to a primary superior lumbar hernia repair

**DOI:** 10.1093/jscr/rjaf386

**Published:** 2025-06-06

**Authors:** Calvin D De Louche, Shoaib Fahad Hussain, Hemant Sheth

**Affiliations:** Department of Surgery and Cancer, Faculty of Medicine, Imperial College London, Praed Street, London, W2 1NY, United Kingdom; Department of General Surgery, Ealing Hospital, London North West University Healthcare NHS Trust, Uxbridge Road, London, UB1 3HW, United Kingdom; Department of General Surgery, Ealing Hospital, London North West University Healthcare NHS Trust, Uxbridge Road, London, UB1 3HW, United Kingdom; Department of General Surgery, Ealing Hospital, London North West University Healthcare NHS Trust, Uxbridge Road, London, UB1 3HW, United Kingdom

**Keywords:** superior lumbar hernia, surgical repair, primary mayo closure, mesh-free closure

## Abstract

Superior lumbar hernias are rare abdominal wall defects that protrude through the superior lumbar triangle and are often misdiagnosed due to their infrequency. This case highlights the successful mesh-free repair of a superior lumbar hernia in a 64-year-old patient, who initially presented with a growing lump and intermittent pain. Though initially suspected to be a sub-fascial lipoma, magnetic resonance imaging was crucial in revealing the true diagnosis. The patient underwent a primary Mayo repair without mesh due to favorable surrounding tissue quality. This report emphasizes the importance of considering lumbar hernias as a differential diagnosis despite their rarity, the potential role of magnetic resonance imaging as an effective imaging modality for diagnosis and surgical planning, and provides strong evidence to support the viability of mesh-free repair techniques in specific cases with good muscle quality.

## Introduction

Superior lumbar hernias are a rare type of posterior abdominal wall hernia that protrude through the super lumbar triangle. Due to their rarity, superior lumbar hernias are often misdiagnosed as more common pathology. As they are subject to no standardized surgical procedural technique, their management is often highly heterogeneous. In this report, we present a case detailing a primary non-mesh repair of a superior lumbar hernia in a 64-year-old male.

## Case report

A 64-year-old gentleman presented with a 3-week history of a persistent left-sided loin to groin pain. He had no history of excessive straining. On examination, the patient displayed 5/5 power in all limbs and no sensory deficit or spinal tenderness. Past medical history included controlled hypertension and asthma, hypercholesterolemia, and glaucoma. A computed tomography scan of the kidneys, ureters, and bladder (CTKUB) returned normal. The patient was subsequently discharged.

Two months later, the patient reported a lump present on the lateral aspect of the left lower back that had grown over the past 6 months. The overlying skin was a normal color, and the lump was intermittently painful. He was referred for ultrasound sonography of his lower back. This was suggestive of a sub-fascial lipoma measuring 3.41 × 1.41 × 4.92 cm with hyperechoic striations. Given the sub-fascial nature of this lesion and recent size increase, a magnetic resonance (MR) scan was recommended to guide surgical excision planning.

**Figure 1:**
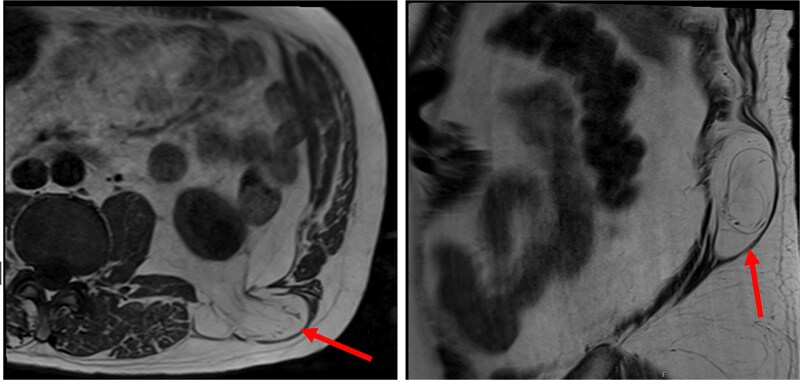
The patient’s MR imaging, which clearly shows the superior lumbar hernia. Axial section (left), sagittal section (right).

At follow-up, the patient was reviewed for consideration of lipoma excision. MR imaging confirmed that the abnormality thought to be a lipoma was in fact a superior lumbar hernia which contained retroperitoneal fat but no bowel or other visceral structure (Fig. 1). The neck of the hernia measured up to 20 mm.

**Figure 2:**
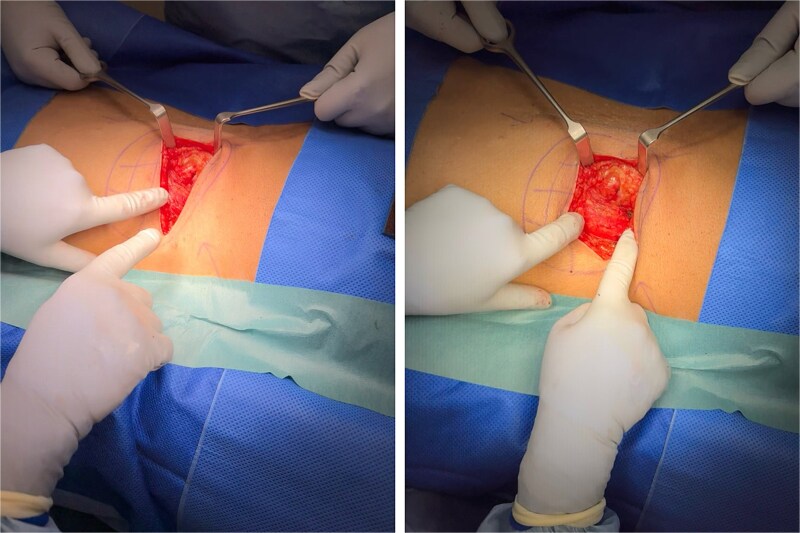
Intraoperative pictures of the superior lumbar hernia.

The patient subsequently underwent an open repair of a left superior lumbar hernia in the prone position. A left paraspinal skin crease incision was made. A single 3 × 3 cm defect was observed at the junction of the left paraspinal and lateral strap muscles (Fig. 2). Fat sheath protrusion and a good muscular sheath were observed, with a narrow hernial neck. The sac and defect were identified, and margins were defined. Excess fat was excised. In view of intraoperative conditions, interrupted sutures were used to complete a primary Mayo hernia repair without mesh. The patient was discharged the following day and recovered uneventfully postoperatively.

## Discussion

We report a case of a superior lumbar hernia repair, which remains sparsely reported within the literature. This case is particularly noteworthy, as it highlights an unusual, yet effective repair technique in the form of a simple primary Mayo closure without mesh in view of good quality surrounding tissues. Previous case reports have comprehensively described lumbar hernia repairs [1–4], but this is one of the only case reports detailing a primary closure of a unilateral superior lumbar hernia without the use of mesh. This case also highlights the importance of considering rare diagnoses in place of more common pathology and the potential for non-traditional imaging modalities to guide surgical management.

Lumbar hernias occur below the 12th rib and above the iliac crest through defects in the lumbar muscles or the posterior fascia [5], and are most commonly found in male patients between the ages of 50–70 [6]. They can be divided into two types, congenital and acquired (primary and secondary), with further subdivision into superior and inferior types occurring in relation to the anatomical location of the hernial neck [5]. Superior lumbar hernias are more common than inferior lumbar hernias [4].

A strong knowledge of the relevant anatomy is essential for management. A superior lumbar hernia (eponymously, a Grynfeltt-Lesshaft hernia) was first reported in 1886 and 1870 [7]. Anatomically, this hernia protrudes through the superior lumbar triangle (triangle of Grynfeltt-Lesshaft) [7]. This is bounded medially by the quadratus lumborum muscle, superiorly by the 12th rib, laterally by the internal oblique muscle, and is floored by the transversalis fascia and the aponeurosis of the transversus abdominis muscle. The external oblique and latissimus dorsi muscles make up the roof [8]. For completeness, inferior lumbar hernias (Petit hernias) occur through the inferior lumbar triangle, and are bounded inferiorly by the iliac crest, anteriorly by the external oblique, posteriorly by latissimus dorsi, and floored by the internal oblique [9].

Clinical presentation in cases like these is often highly heterogeneous, making diagnosis challenging. Misdiagnoses, here as a lipoma, or as other pathologies like abscesses, are therefore common [10, 11]. Nonetheless, a bulge in the lumbar region is commonly observed on examination, which may or may not display impulse on coughing, and reduction when lying laterally. Computed tomography (CT) scans are the gold standard modality for identification of lumbar hernias [6]. However, owing to the initial misdiagnosis, MR imaging was obtained here. Of interest, this was sufficient to aid diagnosis and was adequate for surgical planning. Hence, MR imaging may be considered in future cases, especially with regard to reducing radiation exposure [6].

The cornerstone of managing lumbar hernias has traditionally been via surgical repair, and many techniques have been described, including open mesh repair and the use of advanced muscle flaps [4]. This commonly involves identification of the defect, followed by tension-free siting of a sublay mesh, with a potential muscle flap to overlay the mesh to ensure the defect is fully covered [4]. However, more recently, laparoscopic approaches with mesh application may also be taken, and reports often cite a reduction in reported pain and perioperative morbidity [12].

Here, a primary closure using a Mayo repair [13] without mesh was opted for given the good quality of local tissues which were amenable to closure and to achieve a tension free repair. In these instances, this may be preferable to more established techniques as it is technically simpler, reduces intraoperative duration, and may be more cost effective.

## Conclusion

Superior lumbar hernias are rare but must be considered as a differential when pathognomonic signs are observed in an applicable patient demographic. Non-traditional imaging modalities may be useful in aiding diagnosis and surgical planning. Given the lack of clear consensus regarding best surgical management, individualized surgical repairs, including mesh-free repairs, may be considered in selected cases.
